# The future role of facial image analysis in ACMG classification guidelines

**DOI:** 10.1515/medgen-2023-2014

**Published:** 2023-06-13

**Authors:** Hellen Lesmann, Hannah Klinkhammer, Prof. Dr. med. Dipl. Phys. Peter M. Krawitz

**Affiliations:** University of Bonn, Medical Faculty & University Hospital Bonn Institute of Human Genetics Venusberg-Campus 1 53127 Bonn Germany; University of Bonn Institute for Genomic Statistics and Bioinformatics Bonn Germany; University of Bonn Institute for Genomic Statistics and Bioinformatics Bonn Germany

**Keywords:** variant classification, next-generation phenotyping, phenotypic score, next-generation sequencing, Bayesian statistics,

## Abstract

The use of next-generation sequencing (NGS) has dramatically improved the diagnosis of rare diseases. However, the analysis of genomic data has become complex with the increasing detection of variants by exome and genome sequencing. The American College of Medical Genetics and Genomics (ACMG) and the Association for Molecular Pathology (AMP) developed a 5-tier classification scheme in 2015 for variant interpretation, that has since been widely adopted. Despite efforts to minimise discrepancies in the application of these criteria, inconsistencies still occur. Further specifications for individual genes were developed by Variant Curation Expert Panels (VCEPs) of the Clinical Genome Resource (ClinGen) consortium, that also take into consideration gene or disease specific features. For instance, in disorders with a highly characerstic facial gestalt a “phenotypic match” (PP4) has higher pathogenic evidence than e.g. in a non-syndromic form of intellectual disability. With computational approaches for quantifying the similarity of dysmorphic features results of such analysis can now be used in a refined Bayesian framework for the ACMG/AMP criteria.

## Introduction

Before genetic diagnostics were revolutionised by next-generation sequencing (NGS) technologies, it was the patient’s phenotype that determined which gene would be analysed. The time to establish a molecular diagnosis was therefore strongly dependent on the experience of the examining physician. However, if a variant was found in the gene under investigation, one could be almost certain that this variant was disease-causing.

Over time NGS rapidly transformed clinical medicine and genomics research, pushing the diagnostic yield and time to diagnosis for patients with rare diseases to the next level. With the possibility of examining numerous genes simultaneously, the number of variants that were detected in a single test also increased enormously. With each genome carrying around 3–4 million variants compared to the human reference genome, it became increasingly difficult to decide which of the detected variants actually caused the disorder [8]. This has also altered the way physicians work. With whole exome and genome sequencing, there is less need to start from the patient’s phenotype to choose the right diagnostic method. Rather, the genomic data now has to be evaluated based on the patient’s phenotype [34].

With the increasing acquisition of genomic data and variants, the sharing of data for the assessment of variants is becoming more and more important. Meanwhile through initiatives such as the TRANSLATE NAMSE study (Nationales Aktionsbündnis für Menschen mit Seltenen Erkrankungen) and henceforth the GenomDE initiative, also in Germany, data is now acquired on a large scale [23, 38]. With an increase in data acquisition through genome sequencing instead of exome sequencing, the detection of variants of uncertain significance (VUS) will even continue to increase, making a standardised classification system and efficient prioritisation of genomic data essential. The correct classification is crucial as it will directly affect patient management and determines whether prenatal testing can be offered.

The first attempts to develop a classification approach that distinguishes between “benign” and “pathogenic” were already made in the 1990s [6]. Since then, work has continued on further developing a standardised framework for variant interpretation.

In this review, we would like to describe and discuss the possibilities of implementing phenotypic scores in variant classification guidelines. In recent years, a number of artificial intelligence (AI)-based tools have been developed that are capable of generating phenotypic scores. There are several approaches based on Human Phenotype Ontology (HPO) terms, as well as an increasing number of computer-aided image analysis tools that generate phenotypic scores from medical image data. Since we work on AI-assisted facial image analysis, we will discuss in this article the potential value of such scores in variant classification. We will focus our analysis on phenotypic scores from DeepGestalt, which is the default algorithm in Face2Gene, as well as GestaltMatcher, which is a further development that also supports ultra-rare phenotypes.

## History of Variant Interpretation Guidelines

In 2015, the ACMG and AMP joined forces to provide a recommendation for a general classification scheme based on a survey of laboratories in North America and the existing literature on variant classification [30]. The output of this work resulted in a 5-tier classification scheme that distinguishes between “pathogenic” (P) and “benign” (B) effects. Furthermore, 28 criteria are assigned to the following levels of evidence: very strong (VS), strong (S), moderate (M), supporting (P) or stand-alone (A). After assessment according to these criteria, the variant is classified into the meanwhile widely adopted classes: 1) benign 2) likely benign 3) uncertain significance 4) likely pathogenic and 5) pathogenic. Even though this evaluation scheme is widely adopted by laboratories worldwide as a standardised approach to variant classification, there have been incongruities in the evaluation of variants between different laboratories [2]. It soon became apparent that further specifications were needed for individual genes. Variant Curation Expert Panels (VCEPs) of the Clinical Genome Resource (ClinGen) consortium were formed to develop these disease specifications for the ACMG/AMP guideline. The ClinGen Sequence Variant Interpretation Working Group (SVI) monitors the work of the VCEPs and is responsible for ensuring the uniformity and consistency of the VCEP recommendations [12]. They also publish general recommendations on individual evidence criteria, such as for PVS1 [1], BA1 [10], PS3/BS3 [5] or PP5/BP6 [3].

Another important effort of the SVI was the introduction of a Bayesian framework based on the ACMG/AMP categories, which turned the qualitative into a quantitative approach [36]. The pathogenicity levels (“pathogenic”, “likely pathogenic”, “uncertain significance”, “likely benign” and “benign”) were assigned to posterior probabilities of pathogenicity and the levels of evidence (“supporting”, “moderate”, “strong” and “very strong” as well as “supporting benign” and “strong benign”) translated into odds of pathogenicity modifying the prior probability in a Bayesian approach. In line with the pathogenicity paths defined by ACMG the odds of pathogenicity corresponding to the six evidence levels were then estimated. Using this new approach, it is also possible to combine the pathogenic with benign criteria without necessarily resulting in a VUS and to consider new combinations of criteria that were not described in the original approach [36].

Further refinement of the ACMG/AMP criteria was achieved through the implementation of “Sherloc”, an even more detailed variant classification framework, and the “ABC system”, which, like Sherloc, gives more weight to functional and clinical data [15, 26].

## ACMG – PP4 Criterion

According to the ACMG criteria published in 2015, the PP4 criterion (“Patients phenotype or family history is highly specific for a disease with a single genetic aetiology”) can be used as supporting evidence if the patient has a phenotype for a highly distinctive syndrome. In addition, the gene should not be subject to substantial benign variation and the family history should be consistent with the mode of inheritance of the underlying disease. Obviously, in the presence of only nonspecific clinical features, such as developmental delay, phenotype-genotype correlation cannot be taken as evidence of pathogenicity [30]. However, it is precisely this vague statement that makes a consistent evaluation of this criterion difficult.

Despite the many efforts to minimise incongruences in the application of the classification criteria, they are still frequently applied inconsistently due to subjective influences of the evaluating laboratory [37]. Unlike many other criteria, no specifications have yet been published for the PP4 criterion [20]. Interestingly, based on internal data, a recently published paper revealed that these inconsistencies were mainly caused by inconsistent evaluation of the phenotype – the PP4 criterion [20]. Based on this insight, the authors extended the existing framework “Sherloc” to improve the handling of phenotypic evidence [20, 26]. By curating disease phenotypes that have a high probability of a positive test result in molecular diagnostics for the disease gene, a phenotype diagnostic rate was generated, which can be used to classify the phenotype as either highly or moderately predictive [20].

In 2016 the ACMG criteria were also adopted by the UK Association for clinical genomic science (ACGS) but issued with regularly updated specifications – most recently in 2020 [8]. According to these specifications, a multidisciplinary team (MDT) should evaluate identified variants in relation to the patient’s phenotypic data. In an MDT meeting, it can be decided that the PP4 criterion may also be used as a moderate or strong evidence criterion. For example, if a patient with a *NIPBL* variant has a specific constellation of facial features, severe developmental delay and one of three other criteria (upper limb reduction defects, growth retardation, microcephaly), the level of evidence for the PP4 criterion can be upgraded to moderate. Hunter syndrome and calpainopathy can even be raised to strong level of evidence with appropriate pathognomonic examination results (drug enzyme and muscle biopsy analysis) [8].

However, these attempts to a more objective assessment of the evidence level of the PP4 criterion are also accompanied by several limitations. The approach by Johnson et. al, for example, is highly dependent on the availability of sufficient phenotype data, which is often lacking in reality, as the laboratories usually receive little information on the phenotype. For very rare disorders, this approach is not supported either, since an association of the gene with the genetic disorder must first be proven and sufficient data has to be present for this [20]. Furthermore, the ACGS specialisations are cumbersome, as they would theoretically have to be produced for every gene individually, and some points may again be influenced by the subjective evaluation of the respective clinicians [8].

Therefore, it is long overdue to establish a standardised approach for the application of the PP4 criterion that is as independent as possible from the subjective phenotypic evaluation and that can be implemented in existing evaluation algorithms practicably.

## Next Generation Phenotyping (NGP)

With the increase in the application of NGS, the incorporation of AI in phenotype analysis, has also evolved. In line with NGS, the term next-generation phenotyping (NGP) was formed to reflect the increased need for phenotypic data [14]. In the meantime, software such as Phenomizer exists that can support diagnosis based on the HPO data annotated by clinicians [22, 35]. Beyond the possibility to improve the differential diagnostic process, NGP can also speed up the diagnostic process by being used directly in the prioritisation of exome and genome data [29]. This is often achieved by including Human Phenotype Ontology (HPO) terms in the prioritisation [21, 31, 33].

In recent years, many approaches have also been published which enable the analysis of facial dysmorphisms in patients’ portrait photos and thereby provide suggestions for potential causative disorders [7, 9, 11, 16]. In the past, many published studies on the effectiveness of these approaches have shown that image-based NGP tools can increase efficiency in diagnostics [19, 24, 25, 27]. A query of the number of annotated cases with the HPO term “abnormality of the face” in the Phenomizer, which is based on the OMIM data, revealed that approx. 39% of all listed conditions are labelled with this HPO term. Of course, this can only be used to a very limited extent to say how many of the rare conditions are associated with facial dysmorphism, but the result of this query is also roughly in line with the numbers from previous publications [13]. Patients of these conditions can potentially benefit from phenotypic scores derived from facial image analysis.

GestaltMatcher, for example, an AI that can be seen as a further development of the DeepGestalt algorithm of Face2Gene, provides a phenotypic score for analysed images that delivers a prediction of the extent to which the analysed patient shows similarities to other already diagnosed patients with the suspected disease [16]. The unique advantage of this AI is that, in addition to classification, it also includes cluster analysis, which makes it possible to also analyse and compare syndromes that are not yet associated with a gene [16]. In the following this score will be referred to as GestaltMatcher score.

However, “Prioritization of Exome Data by Image Analysis” (PEDIA) is an approach that integrates portrait photos directly into the interpretation of variants by incorporating phenotypic scores generated by the DeepGestalt algorithm in addition to HPO-based scores [11, 17]. These DeepGestalt scores quantify the similarity of multiple rare phenotypes per individual. Thus, a high DeepGestalt score may facilitate molecular confirmation of the suspected clinical diagnosis by prioritisation [17]. In a cohort of 94 individuals most recently studied in the national framework TRANSLATE NAMSE, the GestaltMatcher score issued by the GestaltMatcher AI was able to improve the prioritisation results with the PEDIA protocol in 86.17% of cases, thus ranking the correct diagnosis higher [23].

NGP can also be used to help decide whether genome sequencing should follow inconclusive previous investigations. This is obviously useful in cases of extremely high phenotypic scores with respect to the general distribution of phenotypic scores without matching findings in molecular genetic testing, as in a recently published case of Koolen de Vries syndrome [4]. If NGP can already be used successfully in these contexts, why not use it also to assist in classifying the pathogenicity of novel variants found in the genome?

Besides the opportunities of NGP, there are also limitations to those approaches. In particular, AI algorithms strongly depend on the quality of the data they are trained on. For rare diseases, only very few images are available to train a face-recognition tool. However, compared to DeepGestalt as a classification approach, GestaltMatcher as a clustering approach has been shown to require significantly fewer training images and, compared to Face2Gene, to significantly increase the performance for ultra-rare disorders [16, 18]. Furthermore, models, including HPO-based scores, will often be trained on human annotated data and will therefore inherit their limitations. The specified phenotype can differ substantially between geneticists. Additionally, predictions will be biased to the human annotations. To account for the variability in assessed phenotypes, it is desirable to collect data from various geneticists and average over their annotations.

### Evidence Level of a Phenotypic Match in the Bayesian Framework

The Bayesian framework representation of the ACMG criteria developed by Tavtigian et al. [30] enables a quantitative assessment of the evidence level of a distinct phenotype via phenotypic scores. As already shown in Johnston et al.[19] and given an adequate experiment, one can estimate the odds of pathogenicity given the observed phenotypic score. Tavtigian et al. transformed the ACMG levels of evidence “very strong”, “strong”, “moderate” and “supporting” into odds for pathogenicity of 350:1, 18.7:1, 4.3:1 and 2.08:1, respectively. As an illustrative example, we show how the ACMG level of evidence of a GestaltMatcher score for a specific syndrome could be assessed. We show results for Cornelia de Lange (CdL) syndrome, Coffin-Siris syndrome and Smith-Magenis syndrome, respectively. Images of all patients are included in the GestaltMatcher Database (GMDB).

### Methods

Using the GestaltMatcher algorithm (version GestaltMatcher-Arc, [18]), the GestaltMatcher score of a patient *i* to a syndrome *S* is given by the greatest cosine similarity to a patient with syndrome *S* (“nearest neighbour”). For *n_train_* patients from syndrome *S* we sampled *n_train_* random patients with other syndromes from the GMDB (covering 321 syndromes with different levels of distinctiveness). We then calculated the GestaltMatcher score to syndrome *S* for all those patients based on the *n_train_* patients from syndrome *S*. Based on the GestaltMatcher scores we conducted a ROC analysis (patients with syndrome *S* = “cases”,random patients = “controls”) to determine a threshold *c* corresponding to the highest Youden index (sensitivity + specificity – 1). For an independent test set of *n_test_* patients from syndrome *S* we then again sample *n_test_* random patients with other syndromes from the GMDB and calculate all GestaltMatcher scores to the first *n_train_* patients from syndrome *S*. The images of the training set were used in the training of the GestaltMatcher algorithm, while the images of the test set were not seen by GestaltMatcher before. We classify the GestaltMatcher scores as “high”, if they fall above the threshold *c*, and as “low” otherwise. Finally, we can estimate the odds of pathogenicity via the positive likelihood ratio given as 
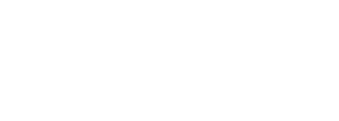
.

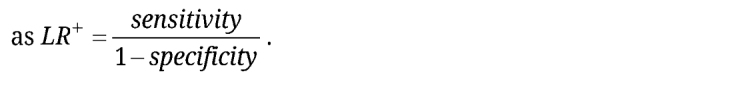



### Results

For CdL 341 CdL patients and 341 random patients of other syndromes from the training data of GestaltMatcher were used to derive a GestaltMatcher score threshold of *c_CdL_* = 0.353 to distinguish between CdL patients and patients of other syndromes. We then applied this threshold to the GestaltMatcher scores of 36 independent patients from CdL syndrome and 36 random patients of other syndromes resulting in contingency table 1.

**Table:** 

	CdL syndrome	Other syndrome
Gestalt score ≥ *c*	32	1*
Gestalt score < *c*	4	36

* For the calculations to work, each field has to be >0 as otherwise we would have a perfect discriminator. Therefore, the observed number of 0 patients in that field has been replaced by 1.

The sensitivity and specificity are then given by

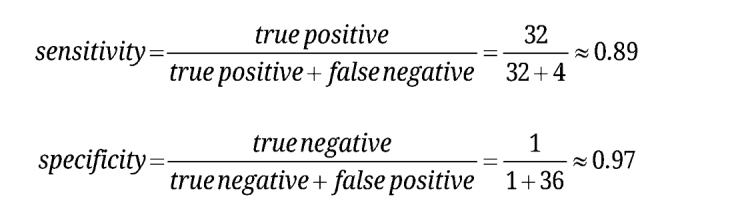




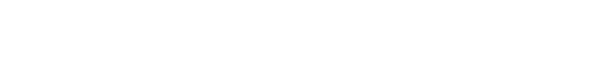



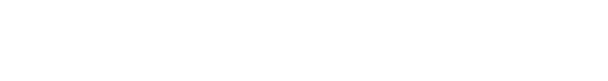


and finally we can estimate the odds of pathogenicity via the positive likelihood ratio given as


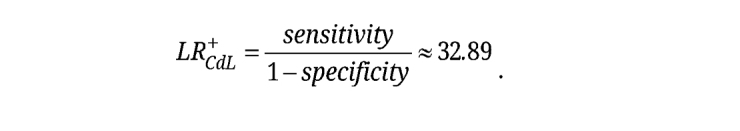



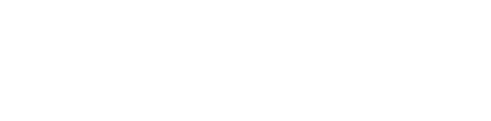
.

This means the probabability that a patient with CdL yields a score above threshold c is roughly 33 times higher than the probability that a patient without the disease will show such a score. In the Bayesian framework of Tavtigian et al., this corresponds to an evidence level of “strong”. For example, a variant in a CdL patient that was submitted to ClinVar last year (NM_133433.4(NIPBL):c.200A>G (p.His67Arg)) – so far – only fulfilled PM2 resulting in a VUS according to the ACMG guidelines. If GestaltMatcher then found a GestaltMatcher score with respect to CdL syndrome greater than *c_CdL_* for that patient, an additional strong criterion would be fulfilled. According to the ACMG guidelines, one strong criterion and one at least moderate criterion give evidence for a likely pathogenic variant (likely pathogenic rule (ii)), i.e. upgrading a previously assigned VUS to a likely pathogenic variant via a phenotypic match.

However, the odds of pathogenicity and therefore the ACMG level of evidence highly depend on the phenotype. For Coffin-Siris syndrome, we used 119 patients with Coffin-Siris syndrome and 119 random patients to derive a threshold of *c_Coffin–Siris_* = 0.318. On a test set comprising additional 11 Coffin-Siris and 11 random patients we found a positive likelihood ratio of = 5 translating into an ACMG evidence level of “moderate”.

**Fig. 1:**
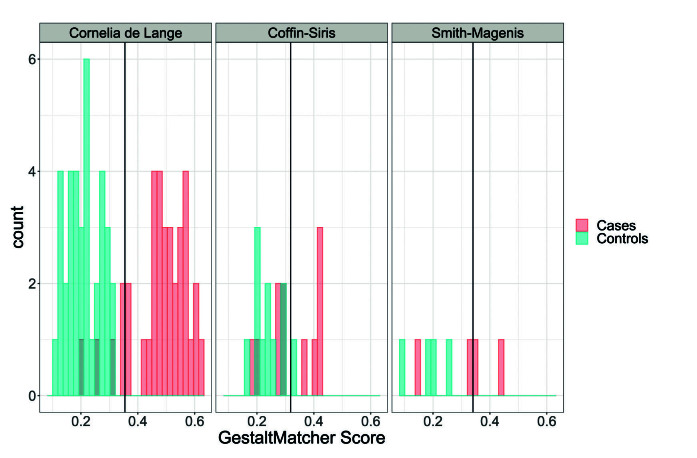
For each of the three illustrative examples, the resulting GestaltMatcher scores on the test set of patients with the named syndrome (“Cases”) and random patients (“Controls”) are shown. The syndrome-specific thresholds *c_CdL_*, *c_Coffin–Siris_*, *c_Smith–Magenis_* are indicated by a black vertical line, respectively.

For a less distinct phenotype as Smith-Magenis syndrome, this approach yielded a likelihood ratio of 2.5 corresponding to an evidence level of “supporting”, i.e. in this case, a phenotypic match would remain in evidence criterion PP4. The threshold for Smith-Magenis syndrome of *c_Smith–Magenis_* = 0.331 was derived from 42 Smith-Magenis patients and 42 random patients and applied on a test set comprising 4 Smith-Magenis patients and 4 random patients. The resulting GestaltMatcher scores of the test sets and thresholds are shown in Figure 1. CdL, which has the highest positive likelihood ratio of the tested syndromes, shows the least overlap between GestaltMatcher scores between CdL patients and random patients, while the derived threshold of Smith-Magenis syndrome does not discriminate well on the test set.

To summarise, these illustrative examples show how GestaltMatcher scores can be translated into ACMG evidence levels. However, they also show that the estimated odds of pathogenicity and therefore the reached ACMG evidence level are influenced by the distinctiveness of the target phenotype as well as by the number of available patients. In these examples, we focused on comparing patients of a syndrome to random dysmorphic patients which corresponds to differential diagnostics. To decide whether a patient is dysmorphic or not, it would be of interest to use healthy controls.

## Conclusion

The application of the PP4 criterion of the ACMG criteria is often prone to subjectivity. Hence, if a facial gestalt is highly distinctive, experienced examiners are more likely to rate the variant as causative [37]. But what is the definition of distinct? The problem is that this estimation is often also dependent on the experience of the examiner. A standardised method to quantify the influence of the phenotype on variant classification more objectively is therefore required. The solution could be the integration of computer-assisted HPO and image analysis into the process of variant classification. Phenotypic scores from AIs such as GestaltMatcher could be used as an influencing factor in the PP4 criterion, which is thus compatible with Bayesian statistics [36]. Besides the contribution to variant classification, GestaltMatcher scores can also be used in variant prioritisation through the direct use of approaches such as PEDIA, which incorporate computer analysis directly into the prioritisation process [17]. This also applies to HPO-based AI tools (e.g. Case Annotations and Disease Annotations (CADA) and Likelihood Ratio Interpretation of Clinical Abnormalities (LIRICAL)), which are likewise interoperable with Bayesian statistics [28, 32]. Approaches like these can also be superior to prioritisation using only HPO terms in cases that exhibit few features other than facial dysmorphism [4].

However, with new technologies constantly evolving, an update of the ACMG/AMP PP4 criterion seems to be long overdue.
